# Ultrastructural and functional fate of recycled vesicles in hippocampal synapses

**DOI:** 10.1038/ncomms9043

**Published:** 2015-08-21

**Authors:** Stephanie A. Rey, Catherine A. Smith, Milena W. Fowler, Freya Crawford, Jemima J. Burden, Kevin Staras

**Affiliations:** 1 School of Life Sciences, University of Sussex, Brighton BN1 9QG, UK; 2 Medical Research Council Laboratory for Molecular Cell Biology, University College London, Gower Street, London WC1E 6BT, UK

## Abstract

Efficient recycling of synaptic vesicles is thought to be critical for sustained information transfer at central terminals. However, the specific contribution that retrieved vesicles make to future transmission events remains unclear. Here we exploit fluorescence and time-stamped electron microscopy to track the functional and positional fate of vesicles endocytosed after readily releasable pool (RRP) stimulation in rat hippocampal synapses. We show that most vesicles are recovered near the active zone but subsequently take up random positions in the cluster, without preferential bias for future use. These vesicles non-selectively queue, advancing towards the release site with further stimulation in an actin-dependent manner. Nonetheless, the small subset of vesicles retrieved recently in the stimulus train persist nearer the active zone and exhibit more privileged use in the next RRP. Our findings reveal heterogeneity in vesicle fate based on nanoscale position and timing rules, providing new insights into the origins of future pool constitution.

## Abstract (web summary)

Synaptic vesicles are efficiently retrieved after transmission but the contribution they make to future signalling remains unclear. Rey *et al.* find that only a subset of vesicles—typically those retrieved recently in the stimulus train—remain near the active zone and exhibit privileged use.

## Graphical abstract

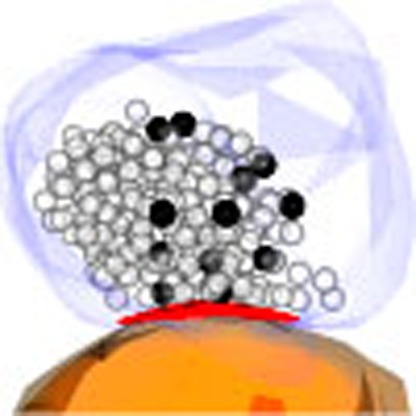

Synaptic vesicles in presynaptic terminals are classified in sub-populations or pools on the basis of function^1^,^2^,^3^,^4^,^5^,^6^. As such, the properties of these pools, their size, physical organization, availability for release and kinetics of recycling, are critical determinants of synaptic performance and of major interest. Arguably the most significant pool class is the readily releasable pool (RRP); by definition, the subset of synaptic vesicles that are first to undergo fusion in response to stimulation^2^,^7^. Thanks to the privileged release status of this pool, and therefore its central relevance in signalling, investigations of the RRP have been the subject of substantial research effort.

In small central presynaptic terminals, recent elegant work based on fast fixation approaches has revealed key steps occurring in the immediate aftermath of vesicle fusion^8^,^9^,^10^. However, the longer-term functional and organizational fate of vesicles reclaimed after a defined pool is accessed, remains poorly understood. For example, in case of the RRP, does the fact that these vesicles undergo preferential use, predispose the retrieved pool to also have privileged release properties? This could be a consequence of a specific characteristic of such vesicles, for example a molecular identifier that earmarks them for selective treatment as suggested for other functionally defined vesicle pools^11^,^12^,^13^,^14^, or perhaps simply reflect the physical positions they adopt after retrieval. Alternatively, such vesicles might exhibit no privileged trafficking or release properties once recycled^15^. A third possibility is that vesicle fate might not be consistent across the retrieved pool, with availability for subsequent use determined according to a non-random pecking order^2^. These issues are of major significance, relevant to fundamental ideas about the relationship between functional and organizational properties of vesicles and the rules that govern their future use.

Here we address the issue of vesicle fate and future pool composition directly, using a combination of sensitive imaging and time-stamped function-ultrastructure approaches in acute hippocampal slices and neuronal cultures. We show that vesicles retrieved after recycling of the RRP are endocytosed close to the active zone but their subsequent fate is variable across the pool. Most vesicles are randomly inserted back into the cluster volume, with no privileged status for further release. We demonstrate that additional synaptic activity and actin remodelling are factors in advancing these vesicles forwards towards the active zone. However, a subset of the retrieved pool becomes preferentially organized at positions near the release site, undergoing privileged use in subsequent signalling events. We investigate the basis for this using reduced stimulation protocols, demonstrating that this subset of the pool correlates with vesicles retrieved most recently in the stimulus train. Our findings reveal a tight link between ultrastructural organization and vesicle function. Heterogeneity in the fate of vesicles retrieved after RRP stimulation provides new understanding of the mechanisms that govern vesicle recycling at size-limited central synapses and the origins of future pool composition.

## Results

### Ultrastructural tracking of endocytosed vesicles

**Figure 1:**
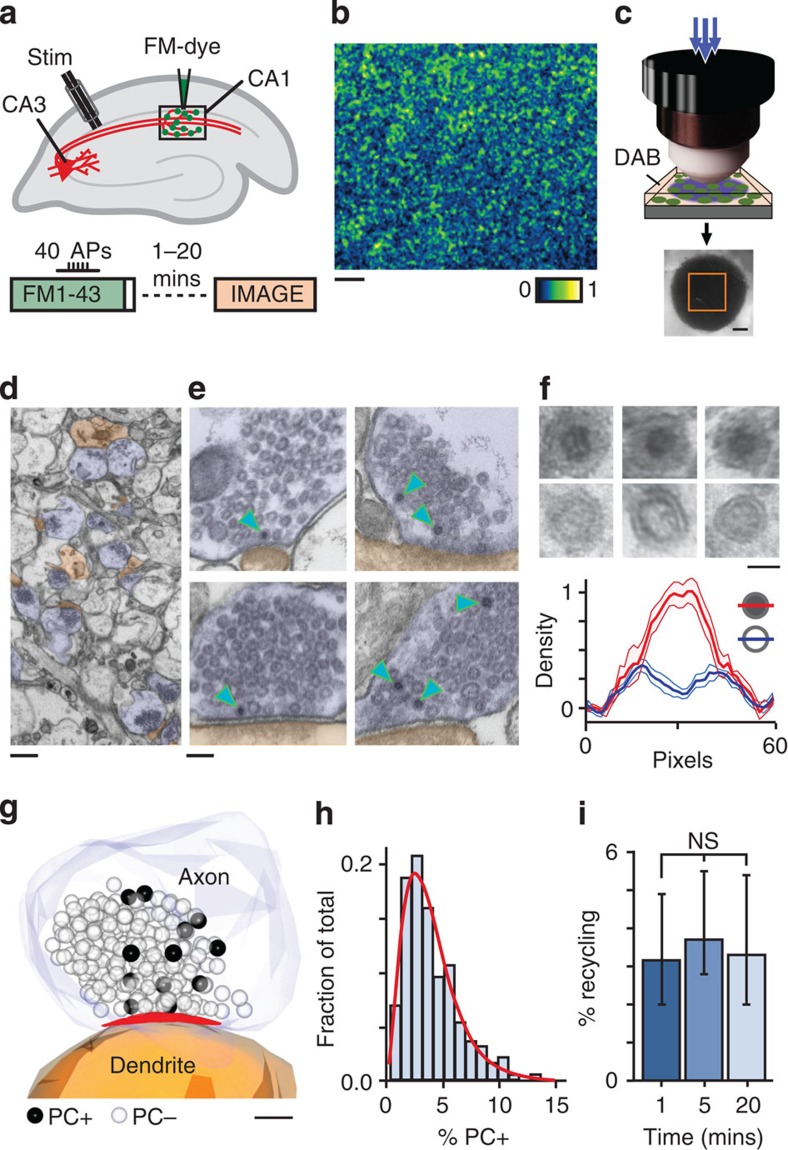
Ultrastructural visualization of vesicles retrieved after RRP stimulation in acute hippocampal slice. (**a**) Schematic illustrating experimental protocol for labelling recycled vesicles. Extracellular stimulation (40 APs 20 Hz) of Schaffer collaterals is combined with FM-dye application to CA1. (**b**) Representative image of fluorescence from dye-labelled synapses in CA1. Scale bar, 10 μm. (**c**) Schematic illustrating approach for photoconversion of labelled vesicles. Dye-loaded fixed target region is photo-illuminated using blue light focused through an objective lens in the presence of DAB (top), producing an electron-dense spot in the slice tissue (bottom). Orange square indicates trimmed target region used for ultrastructural analysis. Scale bar, 200 μm. (**d**) Low magnification electron micrograph showing presynaptic terminals (blue) and postsynaptic structures (brown). Scale bar, 500 nm. (**e**) Typical images showing terminals with PC+ vesicles (arrowheads). Scale bar, 100 nm. (**f**) (Top) High magnification images of PC+ vesicles with electron-dense lumen and PC− vesicles with clear lumen. Scale bar, 25 nm. (Bottom) Cross-sectional density profiles of PC+ and PC− vesicles (*n*=6, mean±s.e.m.) illustrating a quantitative approach that allows for the differentiation of vesicle classes. (**g**) 3D reconstruction showing PC+ vesicles (dark spheres) and PC− vesicles (transparent spheres). Active zone appears red. Scale bar, 100 nm. (**h**) Frequency histogram of PC+ pool sizes based on ultrastructural analysis of *n*=187 synapses from 11 slices from 10 animals, expressed as % of total pool. Red line shows gamma function fit. (**i**) Summary histogram of median±IQR PC+ pool sizes for synapses fixed at different times after loading (1 min: 3.1% (2.0–4.9), *n*=73 PC+ vesicles from 28 synapses (including *n*=8 full serial reconstructions) from 3 slices from 3 animals; 5 min: 3.6% (2.5–5.6), *n*=138 PC+ vesicles from 59 synapses (including *n*=4 full serial reconstructions) from 3 slices from 3 animals; 20 min: 3.3% (2.0–5.4), *n*=455 PC+ vesicles from 100 synapses (including *n*=10 full serial reconstructions) from 5 slices from 4 animals, not significant, Kruskal–Wallis one-way ANOVA, *P*=0.378).

**Figure 2:**
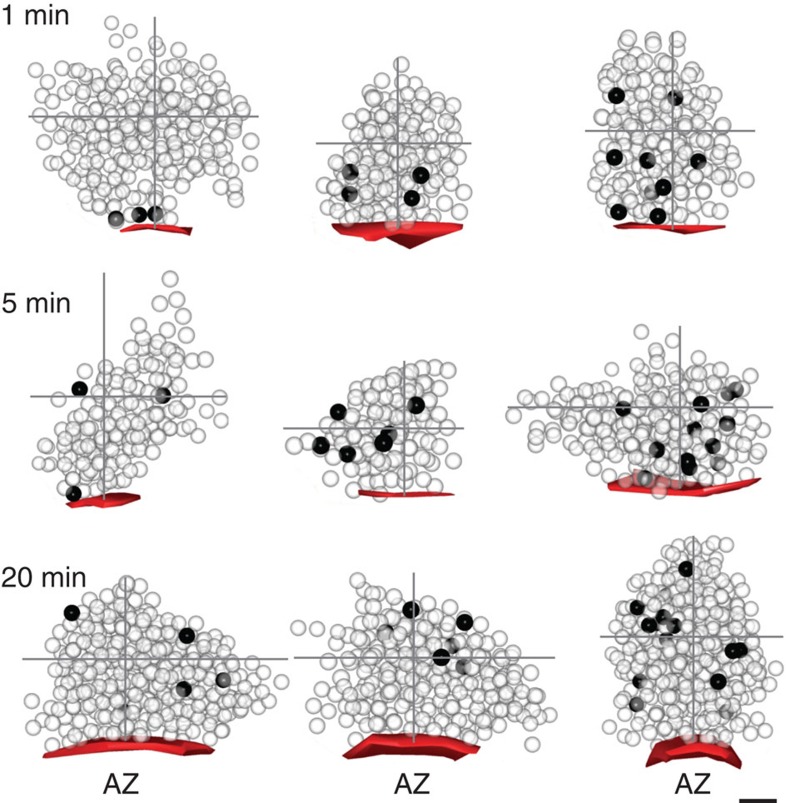
Organization of retrieved vesicle pool at different post-stimulus time points. Representative 3D serial reconstructions for synapses from 1, 5 and 20 min after the loading stimulus. PC+ vesicles are shown as dark spheres and PC− vesicles as transparent spheres. Active zone appears red. Grey cross hairs indicate boundaries of vesicle cluster from active zone centre. Scale bar, 100 nm.

Vesicles recycled after RRP stimulation in acute hippocampal slices were labelled with FM1-43 (refs 16, 17, 18) applied to CA1 while activating Schaffer collaterals (40 action potentials, (APs) at 20 Hz) with field stimulation^19^,^20^,^21^ (Fig. 1a). After loading, confocal imaging revealed discrete punctate staining (Fig. 1b), consistent with dye marking of functional terminals. For ultrastructural investigation we used the capability of FM-dye to drive diaminobenzidine (DAB) polymerization to form an osmiophilic precipitate when photoactivated^8^,^22^,^23^,^24^,^25^,^26^,^27^,^28^,^29^,^30^,^31^,^32^. Dye-labelled brain slices were rapidly fixed at three time points (1, 5 and 20 min) after loading, and the fluorescence-labelled region was photoconverted (Fig. 1c), processed, embedded and sectioned (Fig. 1d^20^,^21^). In ultrastructure, the target area was characterized by presynaptic terminals containing photoconverted (PC+) vesicles with distinctive electron-dense lumen (Fig. 1e,f). Control slices not subjected to activity-evoked FM-dye-labelling or DAB photoconversion or where dye labelling and stimulation were separated in time contained negligible or no PC+ vesicles^21^ and the expression of PC+ vesicles was homogeneous across the target region (Supplementary Fig. 1). We quantified recycled vesicles as a percentage of the total pool using data from serial reconstructions (Figs 1g and 2) and representative central sections. Overall, the retrieved pool size had a broad and skewed distribution (mean±s.e.m. (standard error of mean): 3.9±0.1%; median±interquartile range (IQR): 3.4 (2.2–5.4) %, *n*=455 PC+ vesicles from 187 synapses (including *n*=22 full serial reconstructions) from 11 slices from 10 animals, Fig. 1h). Within this data set, median pool sizes for each time point were very similar (Fig. 1i, quantification in legend) implying that the fluorescence label was stable over time and temporal changes in magnitude were negligible. Our findings provide the first ultrastructural view of the pool recycled after RRP stimulation in small central synapses in acute brain slice.

### Organizational fate of retrieved vesicle pool

**Figure 3:**
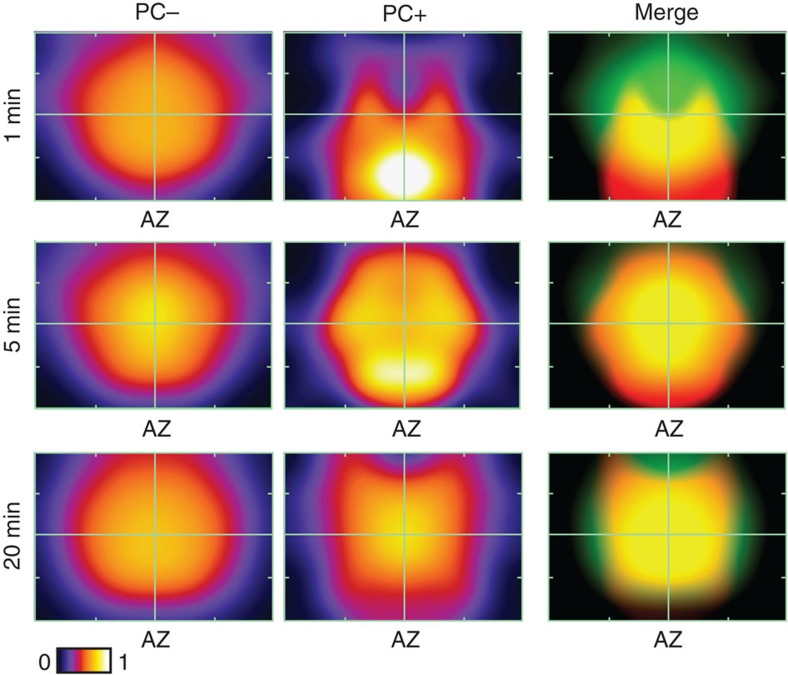
Vesicles retrieved after RRP stimulation recycle to random positions in the terminal. Normalized and smoothed spatial frequency density plots for the three time points (1, 5 and 20 min, *n*=73 PC+ vesicles from 28 synapses from 3 slices, *n*=138 PC+ vesicles from 59 synapses from 3 slices, *n*=455 PC+ vesicles from 100 synapses from 5 slices) for PC− (left panels), PC+ (middle panels) and merged (right panels) showing the positions occupied by vesicles with respect to the active zone and cluster boundaries. Active zone centres (AZ) are at the bottom middle in each plot. In merged plots, green corresponds to PC− and red to PC+ vesicles. See Supplementary Fig. 2 for approach used to generate plots.

**Figure 4:**
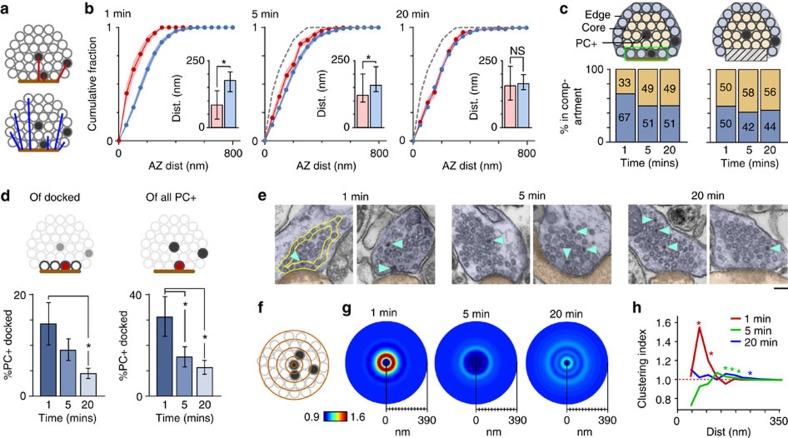
Quantification of recycled vesicle pool spatial dynamics. (**a**) Schematic of distance measure. Filled circles, PC+; empty circles, PC−. (**b**) Cumulative frequency distance plots (1 min, *n*=28; 5 min, *n*=59; 20 min, *n*=100). Lines and circles are mean values and shaded areas are s.e.m. Red lines/circles: PC+, blue lines/circles: PC−. Grey dashed lines: 1 min PC+ data set profile for comparison. Cumulative fraction is fraction of total data set represented by a given distance. (Inset) Median±IQR distance to active zone for PC+ (red) and PC− vesicles (blue). (**c**) Stacked bar charts showing %PC+ vesicles in edge versus core (left) and edge (excluding active zone) versus core (right) with summary schematics. (**d**) Summary of analysis of docked vesicle population. (Left) mean±s.e.m. of PC+ vesicles in pool of docked vesicles as % of total docked vesicle number (mean±s.e.m.: 14.4±4.2%, 9.1±2.1%, 4.5±1.0, *n*=28, *n*=59, *n*=100, ANOVA, *P*<0.01, Bonferroni's: 1 versus 5 min, NS; 5 versus 20 min, NS; 1 versus 20 min, *P*<0.05). (Right) mean±s.e.m. of docked PC+ vesicles as % of all PC+ vesicles (mean±s.e.m.: 31.5±7.9%, 15.5±3.9%, 11.4±2.7, *n*=28, *n*=59, *n*=100, ANOVA, *P*<0.01, Bonferroni's multiple comparisons: 1 versus 5 min, NS; 5 versus 20 min, *P*<0.05; 1 versus 20 min, *P*<0.05). (**e**) Sample micrographs of synapses. PC+ vesicles are marked by arrowheads. Examples of boundaries used to quantify edge and core are illustrated in left panel. Scale bar, 100 nm. (**f**) Schematic of cluster analysis approach based on measuring PC+ fractions in concentric bins around individual PC+ vesicles. (**g**) Circular frequency density plots. Colour look-up table represents fraction of PC+ vesicles. (**h**) Line plots show clustering ratio normalized to ratio for whole synapse. Error bars are omitted for clarity. Asterisks indicate radial distances colour coded for each time point where clustering significantly exceeds baseline values (based on one-sample *t*-tests against 1, see Methods section). NS, not significant.

To examine the positional fate of vesicles recycled after recruitment of the RRP we constructed maps of vesicle organization, referenced to the active zone centre and cluster boundaries for each synapse, and overlaid them to generate smoothed spatial frequency density plots (Fig. 3 and Supplementary Fig. 2). The arrangement of non-recycled (non-photoconverted (PC−)) vesicles was very similar for all time points, confirming that the overall integrity of the vesicle cluster was not influenced by recent activity (Fig. 3, left panels). By contrast, PC+ vesicles had a defined organization that varied with time (Fig. 3, middle and right panels). At 1 min, PC+ vesicles were principally clustered close to the active zone or at sites on the lateral margins of the cluster, compatible with recent retrieval of vesicles adjacent to the release site (Fig. 3, top middle/right). However, at 5 min the labelled pool was homogeneously distributed across the entire vesicle cluster (Fig. 3, middle, middle/right), and by 20 min, PC+ vesicle organization was broadly analogous to the distribution of PC− vesicles (Fig. 3, bottom middle/right). These differences could be directly visualized at the level of individual reconstructed terminals for each time point (Fig. 2). To quantify these observations we measured the Euclidean distance between each vesicle and their nearest point on the active zone (Fig. 4a) using raw non-normalized images. Consistent with the density plots, 1 min PC+ vesicles were significantly closer to the active zone than PC− vesicles (median distance±IQR, PC+: 85 (32–137) nm, PC−: 176 (134–208) nm, *n*=28, Wilcoxon test, *P*<0.001, Fig. 4b, left panel, inset), and characterized by the leftward displacement of the PC+ cumulative frequency plot (red) with respect to the PC− curve (blue). Over time, however, this distinction was lost; the median distance of PC+ vesicles approached that of the PC− vesicles (median distance±IQR, 5 min, PC+: 122 (97–136) nm, PC−: 159 (202–228) nm, *n*=59, Wilcoxon test, *P*<0.001; 20 min, PC+: 156 (104–230) nm, PC−: 163 (142–198) nm, *n*=100, Wilcoxon test, not significant, all 2D (two-dimensional) and 3D (three-dimensional) data combined, Fig. 4b, middle and right panels, inset) and there was a rightward shift of the PC+ cumulative frequency plot (Fig. 4b, middle and right panel). We saw the same pattern if we considered the 2D or 3D data sets alone (Supplementary Fig. 3) validating our use of both types of data sets to increase yield. A compartment analysis based on vesicle positions in electron micrographs confirmed a time-dependent migration of retrieved vesicles from the cluster boundary to take up more central positions in the cluster core at 20 min (Fig. 4c,e). Similarly, we saw a significant decrease in PC+ representation in the population of docked vesicles with time after stimulation (Fig. 4d,e).

Are these temporal dynamics of PC+ vesicles reflected in the positions they occupy with respect to each other? We tested this using a cluster analysis where we calculated PC+ vesicle fractions surrounding each PC+ vesicle at increasing distances from the vesicle centre (Fig. 4f), displayed as circular frequency density plots (Fig. 4g). We observed significant local clustering (60–120 nm) of PC+ vesicles in the 1 min condition (Fig. 4g, left, summary line plot in Fig. 4h) beyond which the fraction tended to baseline levels as the distance radius approached the total synapse size, consistent with spatially limited sites where vesicles accumulate soon after endocytosis^8^,^9^,^10^. At 5–20 min, the distance for significant vesicle clustering extended to >150 nm (Fig. 4g, middle and right, Fig. 4h). Overall, our findings point to a time-dependent dispersal of vesicles retrieved after RRP stimulation, reducing local clustering and leading to non-selective positioning of vesicles within the terminal space.

### Preferential use of a subset of endocytosed vesicles

**Figure 5:**
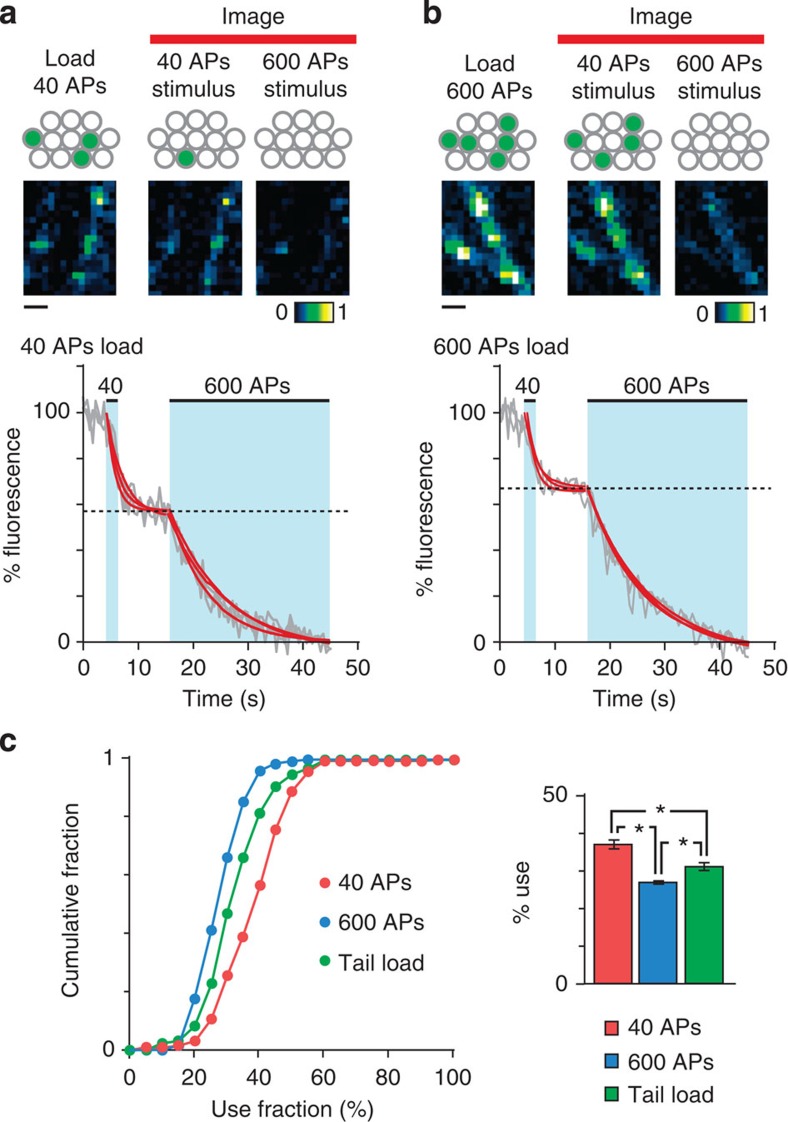
Heterogeneity in functional use of recycled vesicles. (**a**) (Top) Schematic illustrating experimental approach. The pool recycled from recruitment of the RRP (40 APs) is labelled and 20 min later subjected to two rounds of stimulation (destaining) in the absence of dye while imaging; 40 APs to release current RRP and 600 APs to release TRP. The ratio between dye loss after 40 APs stimulus versus total dye loss (after 600 APs) provides a measure of subsequent use of the labelled pool. Note: this is not an absolute measure; although 600 APs and 40 APs should together access >90% of the available pool^15^, residual vesicles or those lost to a non-recycling pool could lead to this value being an overestimate. Accepting these possibilities, normalizing with respect to total release allows comparisons of relative use under different conditions. (Middle) Representative images of 40 APs loading and the two phases of activity-driven dye loss. Scale bar, 2 μm. (Bottom) Plot showing three sample destaining curves (grey lines) each fitted with two single exponentials (red lines). %fluorescence is total fluorescence lost during the two-phase destaining protocol, allowing us to make an estimate of the relative proportion of dye loss arising from the 40 APs versus the larger 600 APs destaining protocol. (**b**) As in (**a**) for 600 APs loading. Scale bar, 2 μm. (**c**) Mean±s.e.m. histogram summary (right) and cumulative frequency distribution plot (left) of % use for 40 APs stimulation after 40 APs loading (*n*=119 from 4 cultures, red) and 600 APs loading in culture (*n*=130 from 3 cultures, blue). Green bar (right) and line plot (left) shows a third protocol (tail load) in which only the tail end (<200 APs) of a 600 APs stimulus train had FM-dye present to allow labelling (*n*=131 from 4 cultures). Mean±s.e.m. use fraction was as follows: 40 APs: 37.2±0.9%, 600 APs: 27.1%±0.6%, tail load: 31.7%±0.9% (one-way ANOVA, *P*<0.001, Bonferroni's multiple comparison: 40 APs versus 600 APs, *P*<0.05, 40 APs versus tail load, *P*<0.05).

If vesicles recycled from an RRP-recruiting stimulation ultimately take up random positions in the cluster, how does this impact on their use during subsequent events that recruit the current RRP? Do these recycled vesicles have privileged fusion capabilities in spite of their non-preferential organization, a finding previously reported in frog neuromuscular junction^28^? We addressed this in time-lapse imaging experiments, examining functional release of the pool previously labelled by RRP stimulation during subsequent rounds of neuronal activity. Specifically we used a protocol in dissociated neurons comprising two steps; an initial 40 APs stimulus to turn over the current RRP followed by a second 600 APs stimulus to release the remaining functional vesicles corresponding to the total recycling pool (TRP, Fig. 5a). In this way, we could examine the relative contribution of labelled vesicles retrieved after RRP stimulation in the current RRP versus their use when the TRP was recruited. The results revealed robust destaining for the 40 APs stimulus, accounting for 37.2±0.9% (*n*=119 from 4 cultures) of total dye loss (Fig. 5a,c). Therefore, a substantial proportion of a pool labelled by a previous RRP stimulus is accessed in an activity protocol that recruits the current RRP. Of course, some level of use should be expected by chance if retrieved vesicles are returned non-selectively to the total recycling pool. To estimate this value we carried out TRP-labelling experiments based on a 600 APs load, where we measured the fraction of the TRP represented by the RRP (Fig. 5b). This yielded a value of 27.3%±0.6% (*n*=131 from 3 cultures), significantly smaller than the use fraction for 40 APs loading (two-tailed unpaired *t*-test, 600 APs versus 40 APs, *P*<0.0001). Thus, vesicles retrieved after release of the RRP are used in a subsequent RRP-recruiting stimulus at a level that significantly exceeds that expected by chance suggesting some privileged release properties. This could relate to such vesicles being specially designated or alternatively reflect some property related to the timing of their retrieval in the stimulus train. To examine this latter idea, we carried out a further protocol in which we biased dye labelling to only the most recently retrieved vesicles in a stimulus train by applying FM-dye at the end (∼200 APs) of a maximal 600 APs loading protocol. In the same two-step destaining paradigm, the fraction of vesicle use for this selective loading of the ‘tail' of the stimulus (31.7±0.87%, *n*=131 from 4 cultures, Fig. 5c, tail load) was intermediate between the 600 APs loading condition and the 40 APs loading protocol (one-way analysis of variance (ANOVA), *P*<0.001, Bonferroni's multiple comparisons: 600 APs versus tail loading: *P*<0.05; 40 APs versus tail loading: *P*<0.05; 600 APs versus 40 APs, *P*<0.05). This implies a significant role for the timing of retrieval in contributing to the subsequent use of those vesicles in the next RRP stimulation.

### Privileged use relates to vesicle position in the cluster

**Figure 6:**
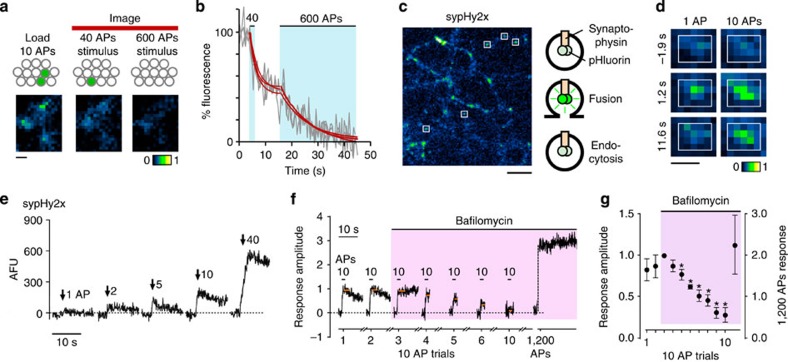
Preferential use of vesicles previously recruited by a limited loading protocol. (**a**) (Top) Schematic illustrating experimental approach. Vesicles that were FM-dye labelled with 10 APs stimulation received two further stimulation steps after 20 min; 40 APs to release current RRP and 600 APs to release TRP. The ratio between dye loss after 40 APs stimulus versus total dye loss provides a measure of preferential use of the original loaded pool. (Bottom) Representative image of 10 APs loading and the two phases of activity-driven dye loss. Scale bar, 2 μm. (**b**) Plot showing sample destaining curves for three synapses each fitted with two single exponentials (red lines). Fraction of 10 APs-loaded vesicles used in subsequent stimulus was significantly higher than 40 APs loading (10 APs: 41.2%±1.6%, 40 APs: 37.2±0.9%, *n*=74 from 7 cultures, *n*=119, two-tailed unpaired *t*-test, *P*<0.05). (**c**) (Left) Example image of sypHy2x expression and schematic illustrating mechanism of action as fusion reporter. Image was collected at 40 AP peak response. White squares show typical small discrete synapses used for analysis. Scale bar, 10 μm. (**d**) Sample sypHy2x images showing fluorescence change at synapse in response to stimulation (1 AP, left; 10 AP, right). Scale bar, 2 μm. (**e**) Traces showing fluorescence intensity profiles for same single synapse from (d) to a range of stimuli. (**f**) Traces showing average fluorescence responses (3 synapses) with repeated 10 APs stimulations (interval 1 min) before (trials 1 and 2) and after (trials 3–10) the addition of 1 μM bafilomycin, a vATPase inhibitor that prevents vesicle re-acidification. Values are normalized relative to the first bafilomycin trial response amplitude. The reduction in response amplitude after bafilomycin treatment is consistent with the use of alkali-trapped vesicles. A larger maintained stimulus (1,200 APs) can recruit additional non-recycled vesicles, observed as a significant rise in fluorescence intensity. (**g**) Mean±s.e.m. responses (105 synapses from 4 cultures), normalized to first bafilomycin trial. Asterisks indicate significant outcomes (*P*<0.05) of two-tailed one-sample *t*-tests for each time point versus 1. Note 1,200 APs response amplitude is shown on different scale (right).

If recent retrieval is an important factor in future use, then a very limited stimulation might be expected to favour the dye marking of vesicles that are predisposed to undergo privileged release. To test this directly we chose a minimal loading stimulus (10 APs, Fig. 6a) that accesses a small vesicle subset representing the most recently recycled vesicles. Consistent with our hypothesis we found that the relative dye loss to the 40 APs destaining stimulus (Fig. 6b, see also Fig. 5a) was significantly larger for this minimal 10 APs loading protocol than for the 40 APs loading condition (10 APs: 41.2%±1.6%, 40 APs: 37.2±0.9%, *n*=74, *n*=119, two-tailed unpaired *t*-test, *P*<0.02, Fig. 6b). This suggests that such a limited loading protocol has a small but significant bias towards recruitment of vesicles that subsequently undergo preferential use.

To provide a further confirmation of this idea we took advantage of the genetically encoded optical reporter sypHy2x^33^,^34^, a fusion construct of synaptophysin and two copies of pHluorin, a pH-sensitive fluorophore that provides a readout of vesicle fusion, endocytosis and vesicle re-acidification (Fig. 6c)^35^. Compared with FM-dye, this probe offers improved sensitivity close to quantal resolution (Fig. 6d,e, see also^34^) and permits readout of multiple recycling rounds at short intervals (Fig. 6f). After generating baseline responses using repeated 10 APs stimulations at 1 min intervals, we added bafilomycin—a v-ATPase inhibitor that serves to alkaline-trap those vesicles that are subsequently turned over^36^. We reasoned that if previously released, now alkaline-trapped, vesicles are used they should not contribute to further fluorescence rises. In this way, we could test if new vesicles were always used on subsequent rounds of 10 APs stimulation, or whether a proportion of the vesicles were the same ones recycled previously. We found that post-bafilomycin, repeated 10 APs stimulations led to progressive reductions in stimulus amplitude (Fig. 6f,g) indicating that larger and larger proportions of the accessed pool were comprised of vesicles that have been recently retrieved. This reduction did not correspond to a depletion of all releasable vesicles since the application of a larger maintained stimulus (1,200 APs) could evoke a substantial additional fluorescence rise (Fig. 6f,g) in the same synapses (Supplementary Fig. 4). Although bafilomycin treatment does cause spontaneous alkalinization, the long time constant of this process (∼60 min^37^) suggests that it is unlikely to be a significant factor in the minute-to-minute timescale changes that we observe. We note also that the magnitudes of each successive amplitude reduction (∼20%), are broadly compatible with what would be predicted on the basis of the previous FM-dye results when scaled down for the smaller 10 AP stimulation protocol used here. These results, consistent with our FM-dye experiments, indicate a small but significant preferential use of the recently retrieved pool.

**Figure 7:**
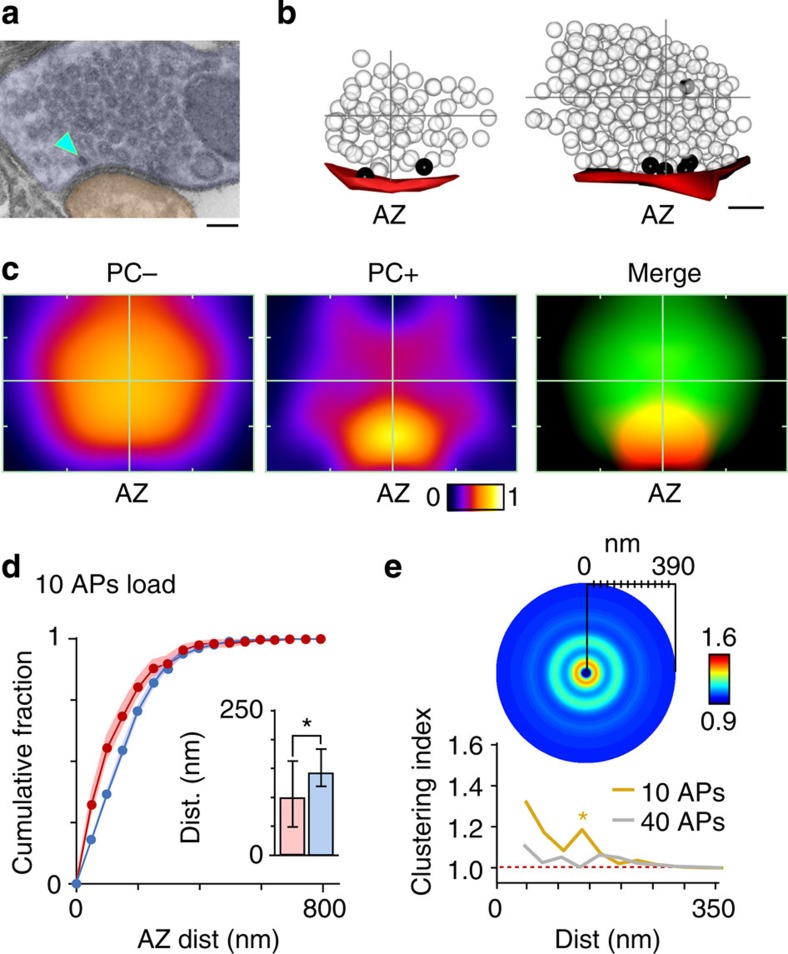
Segregated organization of preferentially used vesicles. (**a**) Representative electron micrograph after 10 APs loading (PC+ vesicle, arrow). Scale bar, 100 nm. (**b**) Representative 3D reconstructions showing PC+ vesicles (dark spheres) and PC− vesicles (transparent spheres). Active zone appears red. Grey cross hairs indicate vesicle cluster boundaries. Scale bar, 100 nm. (**c**) Normalized, smoothed spatial frequency density plots for 10 APs condition (*n*=59) for PC− (left panel), PC+ (middle panel) and merged (right panel). Active zone centres (AZ) are bottom middle in each plot. In merged plots, green is PC−, red is PC+. (**d**) Cumulative frequency plots for vesicle-active zone distances (*n*=59 synapses (including *n*=11 full serial reconstructions) from 3 slices from 2 animals. Lines and circles are mean values and shaded areas are s.e.m. Red lines/circles: PC+, blue lines/circles: PC−. (Inset) Histogram shows median distance±IQR to active zone for PC+ vesicles (red bar) and PC− vesicles (blue bar) (PC+ versus PC−: 99 (50–164) nm, 142 (119–184) nm, *n*=59, Wilcoxon, *P*<0.05). (**e**) (Top) Circular frequency density plot summarizing cluster analysis. Colour look-up table represents fraction of PC+ vesicles. (Bottom) Line plot (orange) showing clustering ratio normalized to ratio for whole synapse. Grey line shows 40 APs 20 min data (from Fig. 4h). Asterisk indicates radial distance where clustering significantly exceeds baseline values (two-tailed one-sample *t*-test, *P*<0.05).

Does our preferentially used subset of vesicles recycled after 10 APs stimulation correspond to the vesicles that are most advantageously positioned in the terminal with respect to the release site? Such a finding would offer a possible structural basis for the observed heterogeneity in fate of retrieved vesicles. To examine the ultrastructural organization of this readily available pool, we carried out 10 APs FM-dye-labelling and then fixed after 20 min for the photoconversion EM preparation outlined above. In ultrastructure, we found that this sub-population of vesicles was highly spatially segregated towards the active zone (Fig. 7a–c), leading to a leftward shift in the PC+ cumulative frequency distribution plot relative to the PC− curve (Fig. 7d), a decrease in the mean distance of PC+ vesicles close to the release site (Fig. 7d, inset) and a rise in the number of PC+ vesicles present in the population of docked vesicles (mean±s.e.m. of docked PC+ vesicles as % of all PC+ vesicles, 10 APs: 21.6±4.740, *n*=59; versus 20 min 40 APs load condition, 11.4±2.7, *n*=100, two-tailed unpaired *t*-test, *P*<0.05). Cluster analysis also revealed a shift towards shorter-range clustering, suggesting an accumulation of PC+ vesicles within a defined sub-compartment of the terminal (Fig. 7e). Our findings support the idea that the vesicles recovered after RRP stimulation are variable with regard to their functional and ultrastructural fates; some vesicles are randomly positioned in the terminal space and undergo non-preferential use but others, in particular those released by a limited stimulus, tend to re-cluster at sites close to the active zone and preferentially participate in transmission on subsequent stimulation.

### Activity advances recycled vesicles towards the active zone

**Figure 8:**
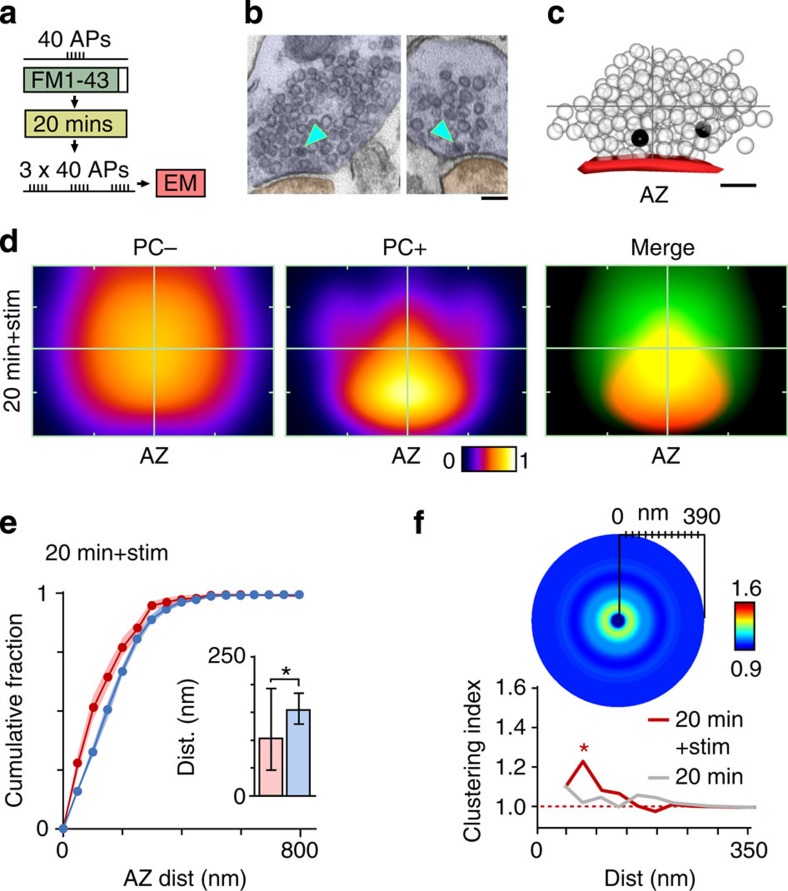
Activity advances recycled vesicles to the active zone. (**a**) Schematic illustrating experimental approach. (**b**) Representative electron micrographs for 20 min+stimulation condition. Scale bar, 100 nm. (**c**) Example of 3D reconstruction for same condition (dark spheres: PC+, transparent spheres: PC−). Active zone appears red. Grey cross hairs indicate boundaries of vesicle cluster from active zone centre. Scale bar, 100 nm. (**d**) Normalized and smoothed spatial frequency density plots for 20 min+stimulation condition (*n*=81 synapses (including *n*=14 full serial reconstructions) from 4 slices from 3 animals) for PC− (left panel), PC+ (middle panel) and merged (right panel) showing the vesicle positions with respect to the active zone and cluster boundaries. Active zone centres (AZ) are bottom middle in each plot. In merged plots, green is PC−, red is PC+. (**e**) Cumulative frequency plots (*n*=81). Lines and circles are mean values and shaded areas are s.e.m. Red lines/circles: PC+, blue lines/circles: PC−. (Inset) Histogram shows median distance±IQR to active zone for PC+ vesicles (red bar) and PC− vesicles (blue bar) (PC+ versus PC−: 104 (46–195) nm, 154 (129–186) nm, *n*=81, Wilcoxon test, *P*<0.0001). (**f**) (Top) Circular frequency density plot showing vesicle clustering, with colour look-up table corresponding to fraction of PC+ vesicles. (Bottom) Line plot (red) showing clustering ratio with increasing distance from vesicle centre, normalized to ratio for whole synapse. Grey line shows 20 min data (from Fig. 4h) for comparison. Asterisk indicates radial distance where clustering significantly exceeds baseline values (two-tailed one-sample *t*-test, *P*<0.05).

Although we established that a subset of vesicles retrieved after RRP stimulation remain close to the active zone for subsequent release, we also show that the majority do not. As such, our findings are different from those previously reported for TRP organization where vesicles preferentially re-cluster at sites near the active zone after recycling^21^. One key difference between RRP and TRP loading protocols is the level of evoked stimulation (40 APs versus >600 APs) suggesting that the additional activity may be an important factor that shapes vesicle organization. To test this idea directly we labelled the vesicles retrieved after RRP recruitment but carried out a further brief stimulation (3 × 40 APs) before ultrastructural processing at 20 min (Fig. 8a). Notably, the remaining non-released fraction of PC+ vesicles had a highly segregated arrangement compared with the standard 20 min condition without stimulation (Fig. 8b–d), with a significantly shorter mean distance to active zone for its PC+ vesicles versus the population of PC− vesicles in the terminal (Fig. 8e, inset, see legend). Moreover, analysis revealed a return to significant clustering of vesicles at a short distance range (Fig. 8f) indicative of a process leading to the condensation of PC+ vesicles into a smaller presynaptic compartment. We also considered whether stimulation might have had some overall impact on the organization of the whole cluster. To examine this we quantified the raw distances of all vesicles to the active zone for the standard 20 min condition (without extra stimulation before fixation), versus the same condition with stimulation. Statistical comparisons revealed a highly significant difference (20 min: median (IQR), 175 nm (98–259), *n*=7,964 vesicles from 100 synapses; 20 min+stim (161 nm (87–244), *n*=7,073 vesicles from 81 synapses), *P*<0.0001, Mann–Whitney test, Supplementary Fig. 5), consistent with the idea that stimulation brings about a small but robust condensation of the whole vesicle cluster towards the active zone.

**Figure 9:**
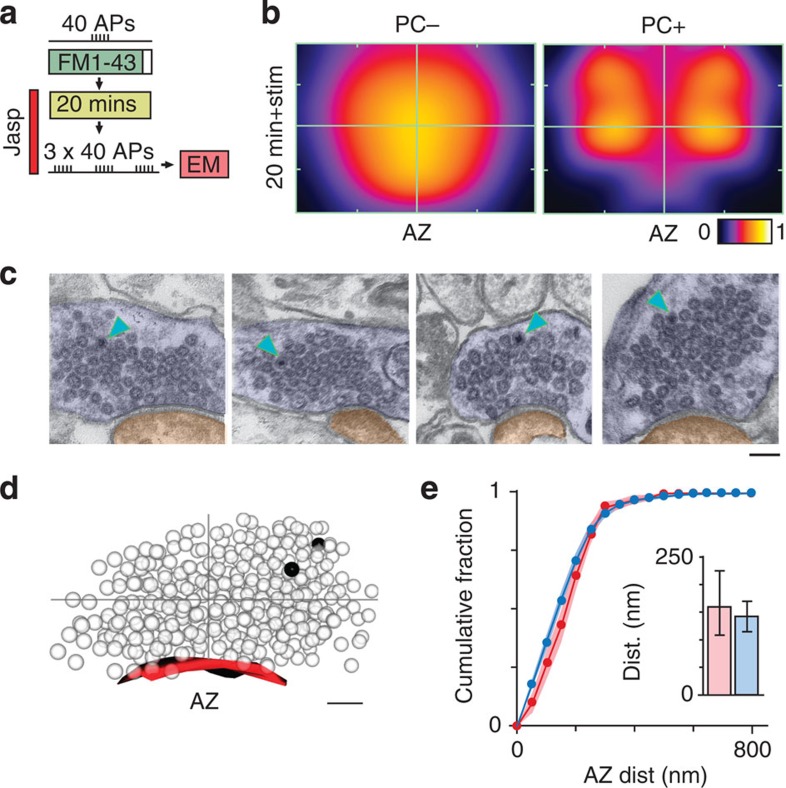
Activity-driven progression of recycled vesicles is inhibited by actin stabilization. (**a**) Schematic illustrating experimental approach. (**b**) Normalized and smoothed spatial frequency density plots for 20 min+stimulation condition in presence of 1 μM jasplakinolide (*n*=48 synapses (including *n*=5 full serial reconstructions) from 3 slices from 1 animal) for PC− (left panel) and PC+ (right panel) showing the vesicle positions with respect to the active zone and cluster boundaries. Active zone centres (AZ) are bottom middle in each plot. (**c**) Representative electron micrographs for same condition. Scale bar, 100 nm. (**d**) Example of 3D reconstruction for same condition (dark spheres: PC+, transparent spheres: PC−). Active zone appears red. Grey cross hairs indicate boundaries of vesicle cluster from active zone centre. Scale bar, 100 nm. (**e**) Cumulative frequency plots (*n*=48). Lines and circles are mean values and shaded areas are s.e.m. Red lines/circles: PC+, blue lines/circles: PC−. (Inset) Histogram shows median distance±IQR to active zone for PC+ vesicles (red bar) and PC− vesicles (blue bar) (PC+ versus PC−: 159 (108–223) nm versus 142 (115–168) nm, *n*=48, Wilcoxon test, *P*=0.090).

We have shown previously that actin is a significant element contributing to the segregation of retrieved vesicles near to the active zone^21^. We reasoned, therefore, that it may also be factor in the stimulation-driven mobilization of vesicles we observe here. To examine this, we incubated 40 AP-loaded slices in 1 μM jasplakinolide, a potent actin stabilizing agent, before the same brief stimulation (3 × 40 APs), fixation and ultrastructural processing steps used above (Fig. 9a). Strikingly, in electron micrographs we observed a robust blockade of the PC+ vesicle segregation at the active zone seen previously (Fig. 9b–d) such that the median distance between the active zone and PC+ vesicles slightly exceeded that for PC− vesicles (Fig. 9e). Moreover, when we quantified the raw distances of all vesicles to the active zone, the condensation effect we previously observed with stimulation was absent (median (IQR), 182 nm (101–267), *n*=3,579 vesicles from 48 synapses; compared with 20 min+stim, *P*<0.0001, Mann–Whitney test).

Taken together, actin appears to have a role in the stimulus-driven mobilization of vesicles—particularly PC+ vesicles situated far from the active zone—and its stabilization strands these vesicles at the rear boundary of the cluster. To examine the functional consequences of this, we expressed sypHy2x and activated neurons with 40 APs stimulation in the presence of 1 μM jasplakinolide. There was only a small decline in response amplitude with repeated rounds of stimulation (2 min intervals)(Supplementary Fig. 6) suggesting that, in the short-term, actin is not a key factor in allowing recruitment of the current RRP to occur. This is consistent with our previous finding that actin inhibition does not block activity-evoked release^21^. However, a large additional stimulus (300 APs) applied between 40 APs bursts significantly accelerated this decline. We suggest that this large activation recycles a significant proportion of the functional vesicle pool, with those near the back of the cluster becoming stranded by actin stabilization, consistent with the ultrastructural data. Thus, in subsequent rounds of RRP stimulation these vesicles are not available for recycling and contribute to the decline in response amplitude. Taken together, these findings support a permissive role for synaptic activation and actin in the participation of recently endocytosed vesicles for subsequent transmission events.

## Discussion

Our results provide a nanoscale view of the fate of the vesicle pool retrieved after turnover of the RRP in small native central terminals, addressing fundamental questions about vesicle use and the origins of the current pool composition. At 1 min after stimulation, we show that recycled vesicles (∼3–4% of the total pool) are significantly segregated near the active zone, compatible with recent findings^8^,^9^. Over longer time periods (5–20 min), however, the overall organization of the retrieved pool changes markedly, with labelled vesicles becoming randomly distributed in the cluster, consistent with their non-selective insertion into the presynaptic terminal volume. On the basis of functional readouts, we show that most vesicles do not exhibit privileged release capabilities after endocytosis and enter the generic recycling pool. Nonetheless, a subset engages in preferential use in a further RRP-recruiting stimulus, significantly exceeding their level of release expected by chance. Using a reduced magnitude loading stimulus (10 APs) we could bias selection towards this subset, demonstrating that they were also physically closer to the release site than the total RRP-recruited vesicle pool. Our results suggest that the long-term fate of vesicles retrieved after RRP stimulation is heterogeneous across the population, with some non-selectively entering the recycling pool while others are effectively fast-tracked for subsequent use.

We make several key assumptions in interpreting our findings. First, in comparing across a variety of different protocols, we have to assume that synapses recruited by different stimuli are homogeneous. We cannot, of course, fully rule out that potential differences among synapses—in some way selectively recruited under different conditions—could account for aspects of the heterogeneous behaviour we observe. However, our evidence based on the fundamental parameters we measure functionally (for example responses evoked by different stimulation protocols using sypHy2x, Fig. 6e, Supplementary Fig. 4) or in ultrastructure (for example, the overall morphology of terminals, their total pool size, the average vesicle distance to the active zone and the number of docked vesicles) indicates that our experiments recruit essentially equivalent synapse types each time. Second, with regard to the interpretation of vesicle dynamics, we make a central assumption that vesicles stay intact after endocytosis (unless they undergo further rounds of release) and thus any subsequent changes in locations at different time points therefore represent time-dependent migration. The fact that the numbers of PC+ vesicles are well-conserved between time points suggests that this is likely to be a reasonable supposition.

What is the basis for this heterogeneity? Our experiments indicate that there is a bias towards subsequent preferential use of the pool retrieved after a minimal stimulation (for example, 10 APs). This effect is not large but nonetheless significant. One possibility is that this protocol favours the turnover of a special subset of RRP vesicles with a characteristic identifier, for example a molecular tag, that singles them out for preferential release in subsequent rounds of stimulation. This would place them in a distinct category where their re-use occurs because of attributes that persist over multiple rounds of activity, an idea that appears at odds with recent findings in the field^9^. An alternative possibility is that vesicle use is a product of their recent endocytosis; the simplest idea being their order of retrieval in the stimulus train with the most recently retrieved vesicles exhibiting higher future use probabilities. In this scenario, stimulating with 10 APs versus 40 APs would favour more use in the retrieved pool in subsequent activity because all such vesicles would, in relative terms, be recently endocytosed. To formalize these ideas, we tested the outcomes of our functional experiments (Supplementary Fig. 7) in a modelled vesicle population with randomly assigned weightings for vesicle use (either heterogeneous or homogeneous across the population). Although we cannot definitively rule out more complex scenarios, a reverse order of retrieval model—effectively ‘last in, first out'—in which vesicles that were most recently endocytosed are favoured for future use, proved to be the most parsimonious solution that matched our experimental findings. Such a mechanism predicts some favoured use of the vesicles retrieved after RRP stimulation, as we observe, without the need for a lingering identifying characteristic of the previously released pool, for example based on molecular determinants that define pool identity^28^,^38^. Such a conclusion is also highly compatible with our ultrastructural findings, which demonstrate that the minimally loaded pool resides near the active zone and is thus presumably advantageously placed to engage in subsequent use. This implies a link between proximity to the active zone and release, consistent with our findings here and those of others^38^.

Our results indicate that a substantial proportion of the retrieved pool of vesicles become randomly distributed in the terminal over 5 and 20 min. What drives this? Recent evidence suggests that intrasynaptic^38^ as well as intersynaptic^23^,^32^,^39^,^40^ movement of vesicles can occur over these timescales, even in resting terminals. Such mobility would presumably be quite effective in dispersing vesicle clusters in the manner we visualize, over tens of minutes. Notably, however, we did not observe such scattering in the case of our limited 10 APs loading over the same time course, perhaps implying that vesicles and associated components lying closest to the active zone remain more effectively anchored. Consistent with this idea, evidence for both a stable active zone-associated core scaffold^41^ and a population of vesicles that are resistant to intersynaptic trafficking^32^ have been described. We also reveal a role for activity in influencing vesicle organization. First, PC+ vesicles exhibited a small but directed progression forwards to the active zone with respect to the cluster volume as a whole. This might be selective for PC+ vesicles but could also be non-selective, perhaps simply a consequence of forces created by the insertion of new endocytosed vesicles at the cluster edges, causing a net movement of resident vesicles forwards. Of course, this stimulus-driven migration of recycled vesicles is subtle, corresponding to a less than one-vesicle-diameter shift in ∼1–2% of the total vesicle pool, observable only thanks to our ultrastructural readout. This may account for the relatively limited role for activity-driven mobilization of vesicles reported in previous studies^42^,^43^,^44^. Moreover, we show that actin contributes to this behaviour; its stabilization with jasplakinolide leaves vesicles stranded near the back with functional consequences that are manifested as a reduction in the released pool size. This aligns well with our previous finding demonstrating that remodelling actin is important in facilitating the movement of the TRP towards the active zone after endocytosis^21^. It remains unclear whether actin directly facilitates vesicle movement or alternatively functions as a support scaffold that can help guide other vesicle-associated elements^45^,^46^. Second, we show that stimulation brings about a small but significant condensation of all vesicles towards the release site and this effect is abolished with jasplakinolide. Previous work has characterized a similar activity-driven condensation of actin towards the active zone^47^ suggesting that these two events could be directly linked.

Heterogeneity in the ultrastructural and functional fate of the retrieved pool offers further insight into previous studies that have considered characteristics of vesicles recycled after stimulation in central synapses. An elegant earlier study by Ryan and Smith^15^ explored the short-term re-use of vesicles after retrieval using fluorescence-based approaches. They considered relatively large labelled pools (100 and 36% of total functional pool) and found no strong evidence for a hierarchy of releasability related to their use history over a timescale of ∼5 min. Our findings would suggest a similar outcome since these significant loading protocols would recycle substantial vesicle populations, and thus mask any small preferential bias related to the most recently retrieved population. Other work has offered an assorted picture of short-term preferential vesicle re-use in central terminals with evidence both in favour^48^,^49^,^50^ and against^37^,^51^. Our long-term ultrastructural tracking of vesicle destiny (over ∼20 min) is not directly comparable to these studies, but the fact that we observe differences in functional vesicle fates according to the nature of the stimulus applied (with limiting stimuli tending to favour more subsequent use and larger stimuli less use), may help to reconcile some of these findings. With regard to vesicle position, elegant studies using comparable time courses to our work have indicated a small^29^ versus marked^38^ segregation of recycled vesicles with respect to the active zone. We note that the loading paradigms reported in these papers (40 APs and 10 APs, respectively) would be highly consistent with our observations of re-clustering according to the level of stimulation. In particular, Park *et al.*^38^ suggest a link between vesicle position in the terminal and the likelihood of release that seems well-aligned with our results. It is notable that our findings contrast with the fate of retrieved vesicles recovered after RRP stimulation in peripheral frog neuromuscular junction. In this large and operationally distinct class of presynaptic terminal, such vesicles exhibited a similar absence of spatial segregation after recycling but displayed robust preferential re-use across the population^28^. As such, physical positioning and release are not highly correlated in this system, and other mechanisms—perhaps physical tracks that link vesicles to the active zone—are more important in preserving RRP status over multiple rounds of recycling^28^.

An interesting consequence of the idea that recent retrieval favours subsequent use can be considered in the case of ongoing low frequency activity. Such a scenario is particularly relevant *in vivo* where spiking frequencies of CA3 pyramidal neurons average ∼1 Hz during behavioural tasks, with only occasional higher frequency bursts^52^. Our findings would predict that, in this case, there should be a tendency towards recycling and use of the same retrieved population of vesicles. As such, a subset considerably smaller than the whole population of functional vesicles at a synapse, would be most relevant to support basal transmission. Consistent with this we note that recent evidence based on ultrastructural assessment of vesicles recycled during basal activity *in vivo*, using a variety of preparation types, reports just such an outcome, with functional pools corresponding to only a very limited fraction (1–5%) of the total vesicle pool^25^.

## Methods

### Acute slice preparation

Experiments were in accordance with the UK-Animal (Scientific Procedures) Act 1986 and satisfied local institutional regulations at the University of Sussex. Acute transverse hippocampal slices (300 μm) were made from 3- to 4-week-old rats (Sprague–Dawley) and maintained in artificial cerebrospinal fluid containing (in mM): 125 NaCl, 2.5 KCl, 25 glucose, 1.25 NaH_2_PO_4_, 26 NaHCO_3_, 1 MgCl_2_, 2 CaCl_2_, 20 μM 6-cyano-7-nitroquinoxaline-2,3-dione, 50 μM AP-5 (bubbled with 95% O_2_ and 5% CO_2_, pH 7.3; refs 19, 30, 32). All experiments were performed at 23–25 °C. FM1–43 or FM1–43FX (Molecular Probes, 20 μM in aCSF) was pressure applied to hippocampal CA1. After 3 min Schaffer collaterals were stimulated at 20 Hz for 2 s (40 APs) or 10 Hz for 1 s (10 APs) using a bipolar tungsten electrode. Imaging experiments were performed 20 min after loading using an Olympus BX51WI microscope (× 60 1 NA objective) equipped with a FV-300 confocal system (Olympus UK), a 488 nm Argon laser and 510 long-pass emission filter. Cells were treated with 1 μM jasplakinolide (Calbiochem) applied immediately after FM-dye-loading.

### Photoconversion and ultrastructural investigation

For ultrastructural analysis, samples underwent rapid microwave fixation (6% gluteraldehyde, 2% formaldehyde in PBS) at 1, 5 or 20 min after dye loading^20^. Subsequently, samples were transferred to 100 mM glycine (1 h), rinsed in 100 mM ammonium chloride (1 min) and washed in PBS. For photoconversion, slices were incubated in an oxygen-bubbled DAB solution (1 mg ml^−1^, Kem En Tec diagnostics). The region of interest, guided by the position of the dye-containing pipette, was illuminated with intense blue light (<500 nm) for ∼40 min. Slices were then washed in PBS, then ice-cold 0.15 M cacodylate buffer, then 0.15 M cacodylate buffer containing 2 mM CaCl_2_, and then prepared for electron microscopy (see ref. 53 for further details). Briefly, a slice was placed on ice for 1 h in a solution containing 3% potassium ferrocyanide (Sigma) in 0.3 M cacodylate buffer with 4 mM CaCl_2_ mixed with an equal volume of 4% osmium tetroxide (Sigma), and then immersed sequentially in filtered warm 1% thiocarbohydrazide solution (Acros Organics) (20 min, room temperature), in 2% osmium tetroxide (Sigma) (30 min, room temperature) and 1% uranyl acetate overnight at 4 °C. After these steps, samples were placed in lead aspartate solution in a 60 °C oven for 30 min. Slices were then successively dehydrated through graded ice-cold alcohols and finally in anhydrous acetone and flat-embedded in Durcupan resin (Sigma) between two ACLAR sheets. Slices were trimmed to the central region of the PC+ region. In depth terms, our previous experiments have demonstrated uniformity in the presence of dye-PC+ vesicles from 2–50 μm from the cut slice surface^20^,^21^. Sectioned samples of 60–70 nm were laid on 300 mesh or formvar-coated slot grids for serial sections and observed using a Hitachi-7100 transmission electron microscope. Digital images were acquired using a 2048 × 2048 CCD (charge-coupled device) camera (Gatan).

### Dye labelling and imaging in primary neuronal cultures

For high-sensitivity readout of functional RRP properties, we used 10–17 day dissociated hippocampal cultures, prepared from P0 Sprague–Dawley rats^23^,^32^. Experiments were performed in HEPES-buffered extracellular solution with composition (in mM): 137 NaCl, 5 KCl, 2.5 CaCl_2_, 1 MgCl_2_, 10 D-glucose, 5 HEPES, 20 μM 6-cyano-7-nitroquinoxaline-2,3-dione, 50 μM AP-5. All experiments were performed at 23–25 °C. Synapses were labelled with field stimulation (10, 40 and 600 APs) in the presence of 10 μM FM1–43 and then washed extensively. 20 min after loading, time-lapse imaging was performed while a two-step FM-dye destaining protocol (40 APs then 600 APs at 20 Hz) was applied. Imaging used an Olympus BX61WI microscope (× 60 1.0 NA dipping objective, excitation: 480/20, emission: 520/35) attached to an Andor Ixon+ electron multiplying charge-coupled device camera (4 × 4 binning, 2 Hz acquisition) controlled by custom-written Micromanager routines. Some experiments used sypHy2x expression based on a viral construct (pAAV-pCAG-sypHy2x-WPRE-bGHpolA, a gift from T. Branco, Cambridge). Neurons were infected after 7 days *in vitro* and used for experiments after a further 9–10 days of expression. Imaging used 4 × 4 binning and 11 Hz acquisition rate. Cells were treated with 1 μM jasplakinolide (Calbiochem) or 0.5 μM bafilomycin A1 (Fisher Scientific).

### Analysis

Images and electron micrographs were analysed using ImageJ (National Institutes of Health), Reconstruct (Synapse Web, http://synapses.clm.utexas.edu/) and custom-written Matlab routines (Mathworks). For ultrastructural data, target synapses with PC+ vesicles were randomly chosen and each vesicle in the terminal classified non-blind either as PC+ or PC− by their vesicle lumenal intensity^23^,^54^. Micrographs were aligned and reconstructed using Xara Designer Pro (Xara) and Reconstruct. Spatial frequency density plots were generated using Matlab routines based on vesicle coordinate positions measured in ImageJ (see Supplementary Fig. 2). Specifically, electron micrographs were oriented with the active zone at the bottom and the coordinates of each vesicle (either PC+ or PC−), as well as the centre point of the active zone, plotted. Synapses with multiple active zones were not analysed. A custom-written Matlab script was used to generate a representation of vesicle positions with lateral symmetry around the midline (see dotted line in Supplementary Fig. 2a) and normalized with respect to the lateral and up-down vesicle cluster boundaries and active zone centre. Note: lateral non-symmetry is not informative because synapses are collected from all orientations in the slice. Normalized maps for all synapses in one condition were overlaid and used to build a 10 × 9 grid density matrix for PC+ and PC− classes, smoothed with a Gaussian filter and colour coded. These plots are used only for visual summaries of vesicle positions; all quantitative analysis of vesicle organization was based on raw non-normalized Euclidean distances from each vesicle to its nearest point on the active zone using the original electron micrographs. A compartment analysis of PC+ vesicle representation in a synapse relied on defining a simple point-to-point shape outlining the cluster, starting from one end of the active zone and returning to the other, tracing the path of the outermost vesicles. Lines linked the outermost vesicles unless the distance between two vesicles was >∼2 vesicle diameters, in which case the line entered the vesicle cluster until it reached the next vesicle. The vesicles in contact with this external boundary line defined the edge vesicle population. A second line, one-vesicle-diameter inside the first line, encompassed the vesicle core population. Visual inspection proved these to be a satisfactory definition of the cluster in each case. Note: the compartment analysis produces a complex polygon that represents the outline of the vesicle cluster; it is not the same as the bounding rectangle used for the generation of spatial frequency density plots.

Fluorescence time-lapse sequences were analysed in ImageJ using raw unfiltered images. For destaining experiments, regions of interest (ROIs, ∼2.9 × 2.9 μm) were identified with the aid of a subtracted image of frames before and after the destaining stimulus, and repeated for both destaining steps. In this way, synapses that underwent destaining in either phase would be included. Chosen puncta were limited to those that were fully encapsulated by the ROI and positionally discrete. For analysis of destaining curves, each profile (4.5 s for 40 APs, 30 s for 600 APs) was fitted with a one-phase exponential decay curve (GraphPad Prism). Curve fitting was constrained to the fluorescence value immediately before stimulation onset. Start and end curve fit values were used to estimate the relative fluorescence loss in each phase.

For vesicle cluster analysis, we measured the fraction of PC+ vesicles in circular ROIs of increasing size (50 nm radial distance steps) surrounding individual PC+ vesicles. All values for these circular bins were then normalized to the fraction of PC+ vesicles in the synapse achieved when the bin size encompassed the total vesicle cluster; for this reason the right hand end of all plots was 1. Normalizing to 1 in each case allowed for comparisons within a condition, and for plots to be compared across conditions. Values at each distance were tested for significance against 1 using one-sample *t*-tests to establish whether local clustering was significantly different (higher or lower) than the overall (baseline) level of clustering of PC+ vesicles in the terminal.

Statistical comparisons used GraphPad Prism. Data sets were tested for normal distribution using the D'Agostino–Pearson test. Where data sets did not conform we reported median and IQR and applied non-parametric comparisons (Wilcoxon tests for paired, Mann–Whitney tests for unpaired). For parametric comparisons we reported mean±s.e.m. and tests between multiple groups used one-way ANOVA with post-hoc Bonferroni's multiple comparison tests. Two sample comparisons used unpaired or paired *t*-tests. One-sample *t*-tests were used for comparing a sample to a single value. Significance was defined as *P*<0.05.

## Additional information

**How to cite this article:** Rey, S. *et al.* Ultrastructural and functional fate of recycled vesicles in hippocampal synapses. *Nat. Commun.* 6:8043 doi: 10.1038/ncomms9043 (2015).

## Supplementary Material

Supplementary Information Supplementary Figures 1-7
