# Supplementary material for: Ultrastructural and functional fate of recycled vesicles in hippocampal synapses
Source: Nat Commun. 2015 Aug 21;6:8043. doi: 10.1038/ncomms9043 (PMC4560786; doi:10.1038/ncomms9043)
Supplement: Supplementary Information — Supplementary Figures 1-7 [file ncomms9043-s1.pdf]

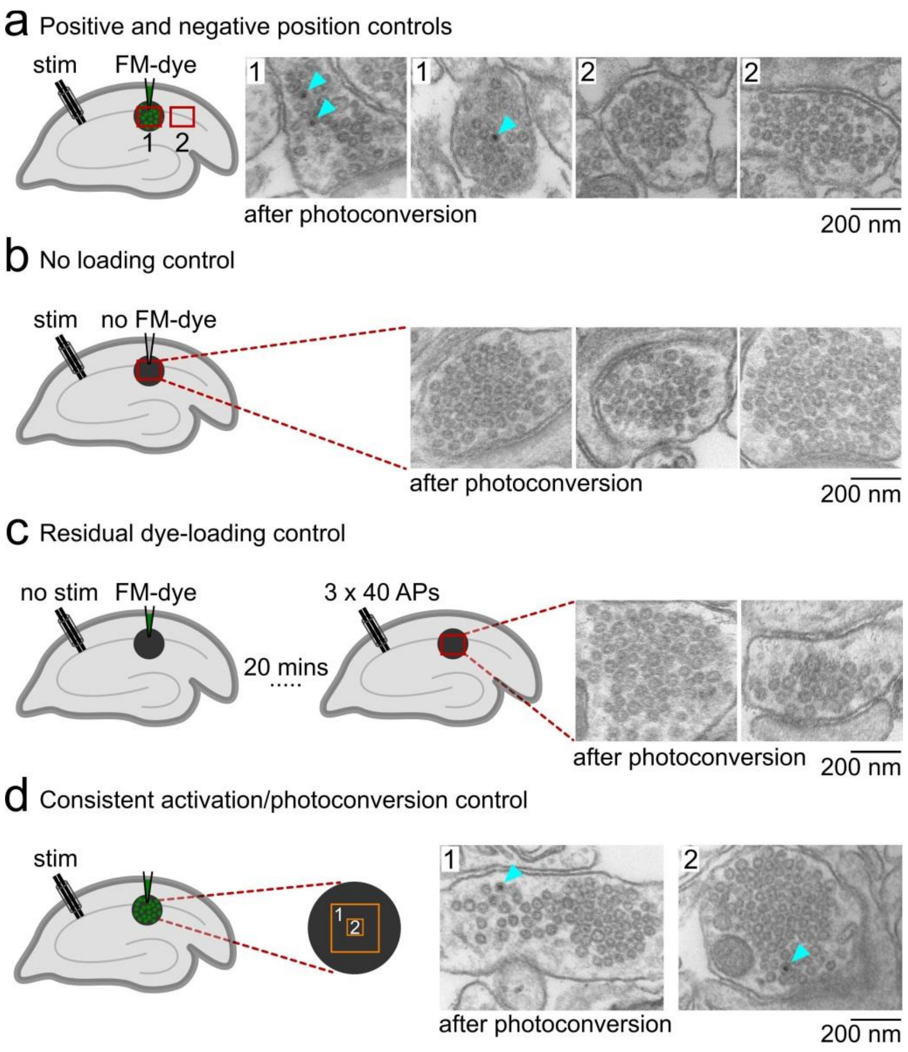

Supplementary Figure 1. Controls for photoconversion experiments. (a) Positive and negative position controls. Slices labelled and photoconverted have PC+ vesicles in target area (1) but not in a laterally displaced region where no labelling or photoconversion took place (2). Three sections (from 1 slice from 1 animal) were systematically scanned, examining >1000 terminals. **(b)** No FM-dye-loading control. Slices stimulated in the absence of FM-dye and then subjected to the normal photoconversion process have no PC+ vesicles. Three sections (from 3 slices from 2 animals) were systematically scanned, examining >1000 terminals. **(c)** Residual dye-loading control. We tested whether any residual dye that remained on the extracellular surface after washing could label vesicle populations recycled at a later stimulation timepoint. We found negligible numbers of photoconverted vesicles (median ± IQR (interquartile range): 0.0 [0.0-0.0] from 259 synapses from 2 slices from 1 animal) **(d)** Consistency of photoconversion control. The target region is a ~500 x 500 μm area inside the boundaries of the photoconversion site (outer orange square). To assess consistency of stimulation, dye-labeling and photoconversion across this region we measured vesicle fractions at the region centre ('2') versus peripheral regions ('1'). The appearance of PC+ vesicles was equivalent in both regions and fractions were not different (median ± IQR (interquartile range), region 1: 3.45 [2.44-5.57] from 21 synapses; region 2: 3.03 [1.55-4.60] from 18 synapses from 1 slice from 1 animal, Mann-Whitney test, P = 0.31, not significant).

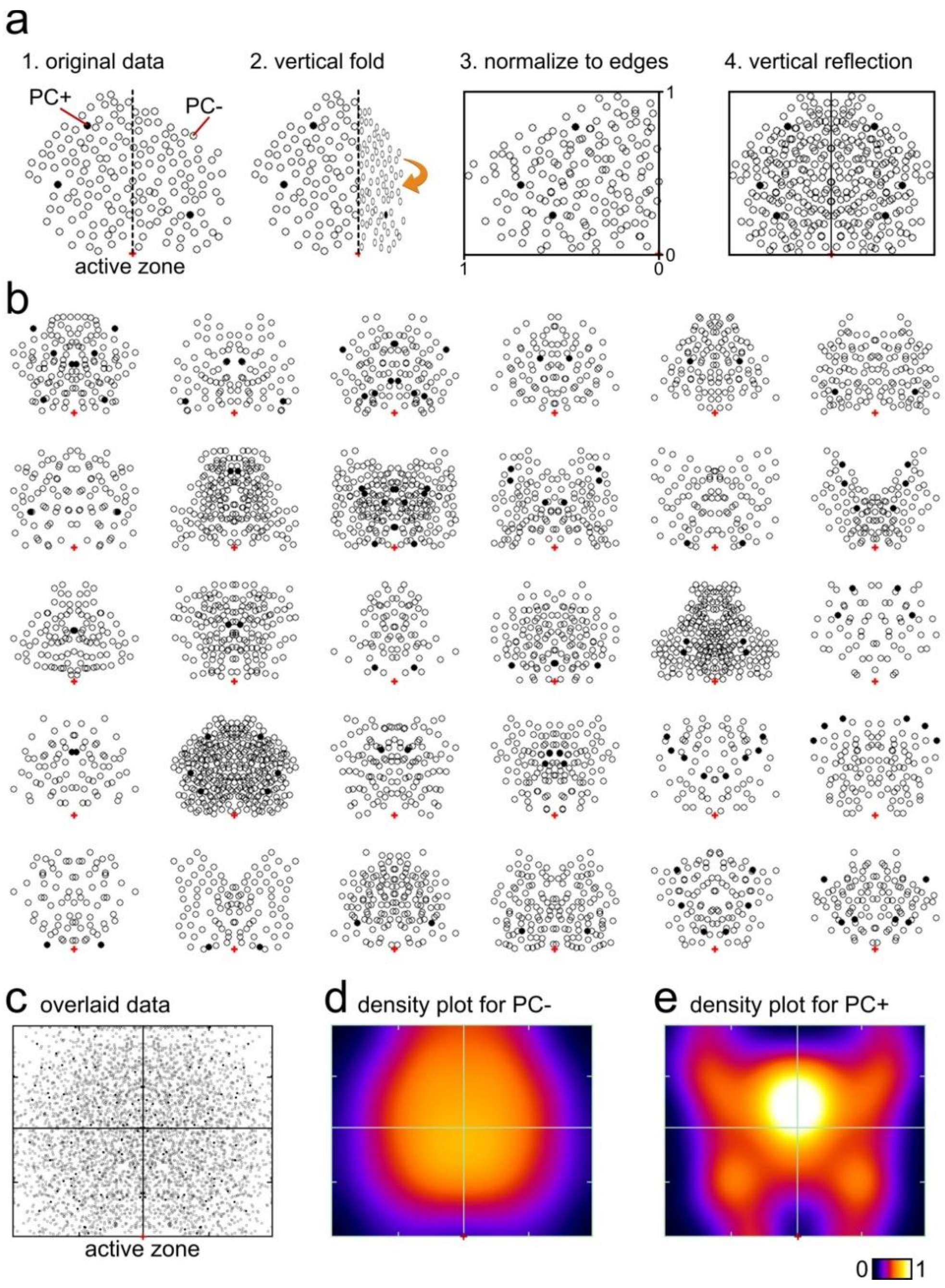

Supplementary Figure 2. Approach for generating spatial frequency density plots. (a) 1. Cartoon of vesicle distribution for a typical synaptic profile. Filled circles, PC+ vesicles; Empty circles, PC- vesicles. Active zone centre is marked with red cross. 2. Left-right asymmetry is removed by folding one half onto the other around centre line defined by active zone. 3. Vesicle coordinates with respect to active zone are normalized to bounding edges. This allows us to plot each vesicle position in grid coordinates with respect to the centre of the active zone and the cluster extremes. 4. All vesicles are reflected around centre line. **(b)** Example schematic representations of 30 single sections from ultrastructural data as in a4. **(c)** Summary plot of vesicle coordinates from (b) overlaid to produce a cluster map for each vesicle class. **(d,e)** Spatial frequency density plots for PC- **(d)** and PC+ **(e)** are generated by calculating the relative density of vesicles in each square of a 10 x 9 grid that encompasses the synapse area and representing them with a colour look-up-table smoothed with a simple Gaussian filter.

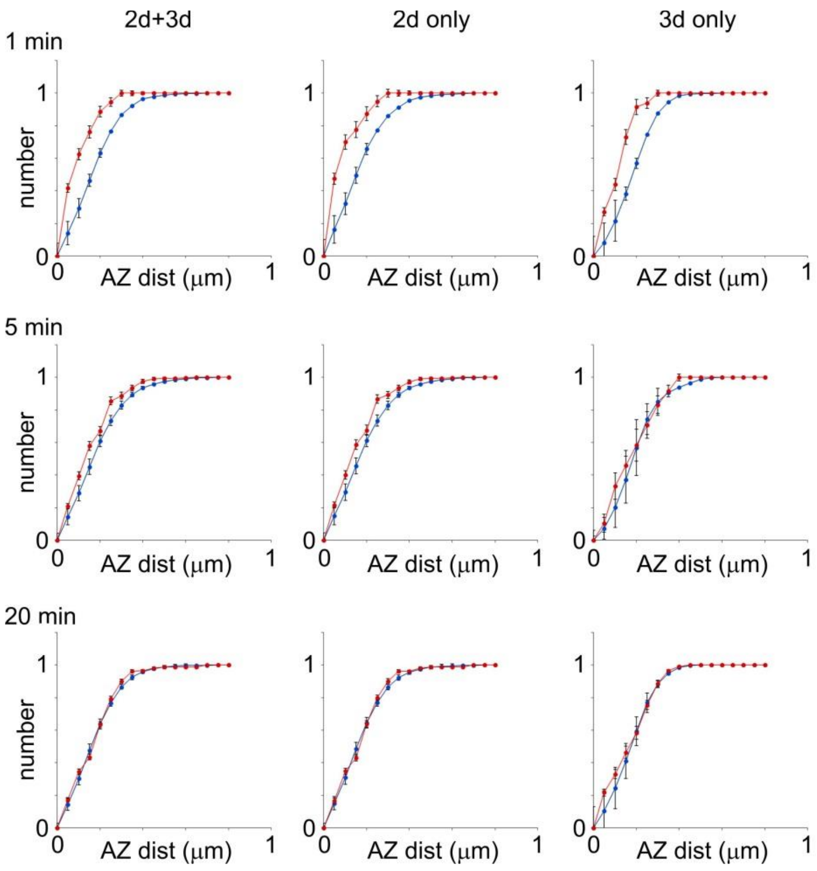

Supplementary Figure 3. Vesicle positions measured for 2d versus 3d datasets yield analogous results. Summary of cumulative distance plots (mean ± SEM) for combined 2d+3d (1 min: n = 28, 5 min: n = 59, 20 min: n = 100), 2d only (1 min: n = 20, 5 min: n = 55, 20 min: n = 90) and 3d only (1 min: n = 8, 5 min: n = 4, 20 min: n = 10) datasets yield analogous results for each condition. Red lines/circles, PC+; blue lines/circles, PC-. PC-versus PC- distances to active zone for 2d versus 3d, 2d versus 2d+3d, 3d versus 2d+3d, 1 min, 5 min, 20 mins, P > 0.271 - 0.9884, unpaired comparisons (Mann-Whitney tests), all comparisons not significant. PC+ versus PC+ for 2d versus 3d, 2d versus 2d+3d, 3d versus 2d+3d, 1 min, 5 min, 20 mins, P > 0.361 - 0.903, unpaired comparisons (Mann-Whitney tests), all comparisons not significant. Paired comparisons (Wilcoxon tests) of PC+ versus PC- distances to active zone, 1 min 2d, P < 0.001 (n = 20, 20), 1 min 3d, P < 0.0078 (n = 8, 8), 1 min 2d+3d, P < 0.0001 (n = 28, 28). PC+ versus PC-, 5 min 2d, P < 0.016 (n = 55, 55), 5 min 3d, P = 0.125 (n = 4, 4), 5 min 2d+3d, P < 0.01 (n = 59, 59). PC+ versus PC-, 20 min 2d, P = 0.46 (n = 90, 90), 20 min 3d, P = 0.77 (n = 10, 10), 20 min 2d+3d, P = 0.42 (n = 100, 100).

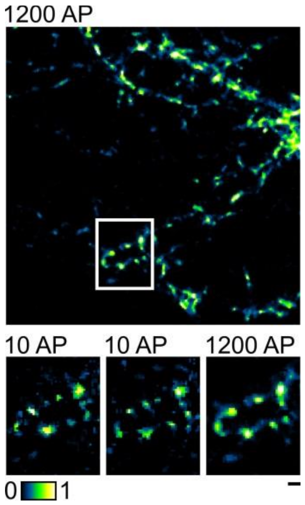

Supplementary Figure 4. Different stimulation protocols activate the same synaptic population. (Top) SypHy fluorescence image showing response to 1200 AP stimulation. Image is generated by subtracting pre-stimulus baseline images from stimulation peak images to reveal activated synaptic terminals. (Bottom) Same analysis on region shown in white rectangle (top) for 10 AP pre-bafilomycin (left), 10 AP post-bafilomycin (middle), and 1200 AP (right) responses. The patterns of activated synapses are essentially identical in each case, showing that the same terminals are recruited. Note: 1200 AP fluorescence image is intensity-scaled differently to 10 AP responses to avoid an over-saturated appearance. Scale bar, 2 μm.

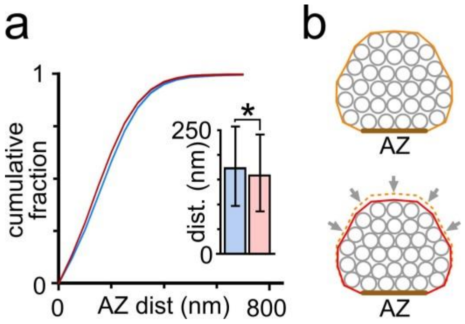

Supplementary Figure 5. Condensation of total vesicle cluster towards active zone with stimulation. (a) Cumulative plot showing distance to nearest point on active zone for all vesicles for 20 minute condition (blue) and 20 minute + 3 x RRP condition (red). Statistical comparisons revealed a highly significant difference (20 min: median [IQR], 175 nm [98-259], n = 7964 vesicles from 100 synapses [including n = 10 full serial reconstructions]; 20 min + stim (161 nm [87-244], n = 7073 vesicles from 81 synapses [including n = 14 full serial reconstructions]), P < 0.0001, Mann-Whitney test), suggesting that stimulation brings about a small but robust condensation of the whole vesicle cluster towards the active zone. **(b)** cartoon illustrating phenomenon before (top) and after stimulation (bottom).

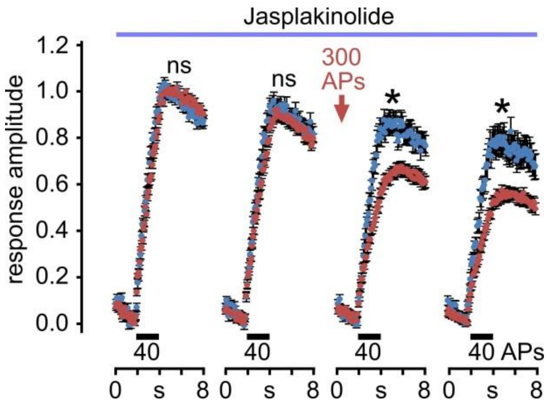

Supplementary Figure 6. Evidence for actin-dependency in retrieved vesicle use. Mean ± SEM fluorescence intensity traces for SypHy2x-expressing synapses treated with 1 μM jasplakinolide and stimulated with 40 APs every 2 minutes. In the red trace, an additional 300 APs is applied between the 2nd and 3rd responses causing a significant additional reduction in response amplitude compared to the unstimulated blue control trace (value at peak for 3rd response, control: 0.85 ± 0.04, n = 86 synapses from 3 cultures, stimulated: 0.62 ± 0.02, n = 138 synapses from 3 cultures, two-tailed unpaired t-test, P < 0.001, asterisk; value at peak for 4th response, control: 0.76 ± 0.06, n = 86 synapses from 3 cultures, stimulated: 0.51 ± 0.02, n = 138 synapses from 3 cultures, two-tailed unpaired t-test, P < 0.001, asterisk).

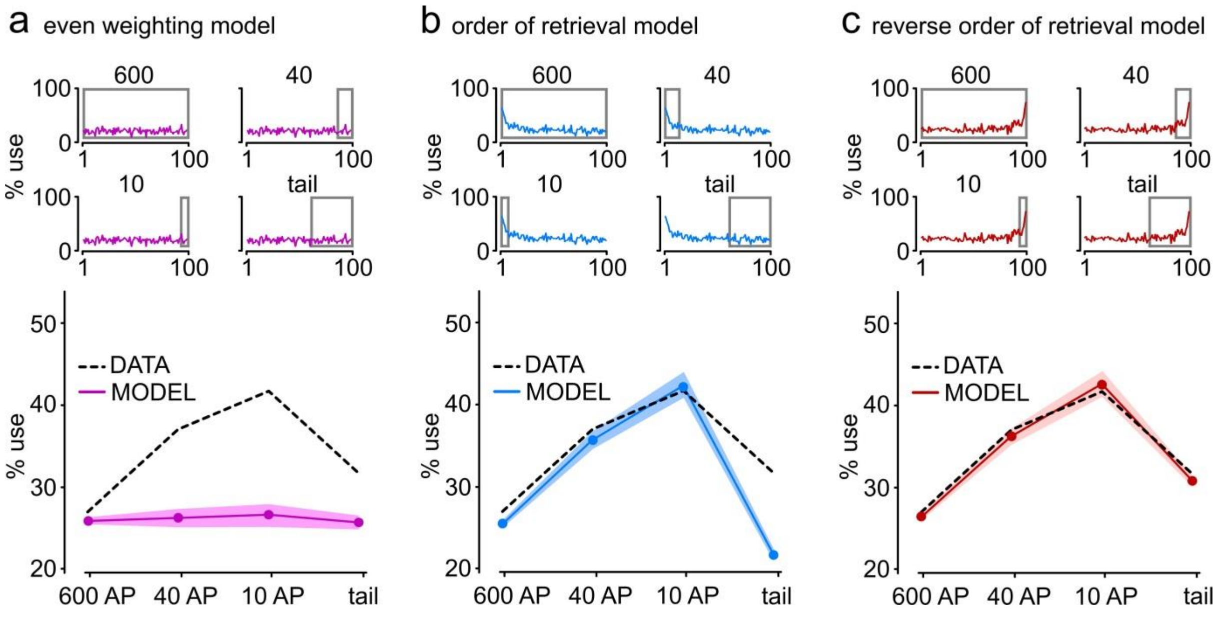

Supplementary Figure 7. A simple compartmental model of future vesicle use. To explore possible scenarios of future vesicle use, we constructed a simple model based on 100 functional vesicles assigned a random future use probability with different weightings. The weightings define alternative rules that could influence subsequent use behaviour. In **(a-c)** the top panels show the weighting profile across the vesicle population for each of the three models, generated from the average of 100 random trials. In each trial, each vesicle is randomly assigned a 0 or 1 value (no use/use) with the outcome probabilities weighted according to the model being considered. Grey rectangles indicate the vesicle population recruited for each of the stimulation conditions (see below). The main plots indicate the % use to a 40 APs stimulus versus a 600 APs stimulus consistent with the experimental protocols used in Figs. 5 and 6. Lines and shaded regions are mean ± SEM from 100 replicate trials for the four experimental conditions (600 APs load, 40 APs load, 10 APs load, and 'tail' load). Actual data is indicated by dashed black lines in each case. In all scenarios, total weighting is the same across the 100 vesicles, corresponding to the retrieval of ~27% of the pool for subsequent use in the case of the maximal load (600 AP) condition, consistent with the experiment data. **(a)** Even weighting model. All vesicles have equal chance of being preferentially used in subsequent rounds of stimulation. **(b)** Order of retrieval model. Vesicles recycled at the start of the stimulus train have privileged use properties, compatible with a special status for readily-released vesicles. **(c)** Reverse order of retrieval model where most recently retrieved vesicles in the stimulus train have preferential use characteristics. This model shows excellent agreement with the experimental data.
